# Integrated liquid biopsy model for predicting metastasis and guiding PD-1 therapy in esophageal squamous cell carcinoma

**DOI:** 10.3389/fonc.2025.1673946

**Published:** 2025-11-26

**Authors:** Hailong Pang, Shenshen Hao, Shuiwang Hu

**Affiliations:** 1 Department of Clinical Laboratory, General Hospital of China Pingmei Shenma Medical Group, Pingdingshan, China; 2 Department of Spinal Oncology, General Hospital of China Pingmei Shenma Medical Group, Pingdingshan, China; 3 Guangdong Provincial Key Laboratory of Proteomics, Department of Pathophysiology, School of Basic Medical Sciences, Southern Medical University, Guangzhou, China

**Keywords:** esophageal squamous cell carcinoma, lymphnode metastasis, liquid biopsy, integrated biomarker signature, PD-1 inhibitor

## Abstract

**Objective:**

This study endeavors to develop and validate an integrated biomarker signature (IBS) grounded in serum carbohydrate antigen 72-4 (CA72-4), vascular endothelial growth factor-C (VEGF-C), and the pepsinogen I/II ratio (PGI/PGII). A practical IBS model will be constructed to substantially enhance the accuracy of lymph node metastasis (LNM) risk stratification following surgery for esophageal squamous cell carcinoma (ESCC). This model is anticipated to refine prognostic assessments for patients and to identify novel research avenues within the field, thereby providing guidance for both prognostic determination and future investigations.

**Methods:**

A prospective three-cohort design was adopted, encompassing a training cohort of 220 patients, a temporal validation cohort of 138 patients, and a regional external validation cohort of 94 patients. This design was selected for its robustness in ensuring the validity and reliability of the findings. A total of 452 patients with esophageal squamous cell carcinoma (ESCC) who underwent R0 resection were enrolled between March 2022 and June 2024. The predictive model was constructed using the XGBoost algorithm combined with Shapley Additive exPlanations (SHAP) for interpretability. Model performance was evaluated via receiver operating characteristic (ROC) curves, decision curve analysis, and net reclassification index.

**Results:**

The IBS model demonstrated superior discriminative ability in the training cohort (*n* = 220; area under the receiver operating characteristic curve (AUC) = 0.936; 95% confidence interval (CI): 0.908–0.964) compared to the AJCC ninth edition staging system (ΔAUC = 0.154; *p* < 0.001). Performance was maintained in validation cohorts. High-risk patients (IBS > 0.5) receiving PD-1 inhibitor plus chemotherapy achieved a pathological complete response (pCR) rate of 28.6%, representing a 115% increase over conventional therapy (*p* = 0.009).

**Conclusion:**

The validated IBS model provides high-precision prediction of postoperative LNM risk in ESCC. It offers a novel framework (**“**tumor antigen burden–lymphangiogenesis–immune microenvironment**”**) for improving patient risk stratification, guiding adjuvant therapy decisions (including immunotherapy response prediction), and optimizing resource allocation, thereby potentially impacting ESCC management paradigms.

## 1 Introduction

Esophageal squamous cell carcinoma (ESCC) constitutes approximately 85% of global esophageal cancer cases and poses a significant health burden, particularly in East Asia, where over 80% of the estimated 647,000 new cases in 2024 are expected to occur (1–3). Despite advances in multimodal therapy incorporating radical surgery and neoadjuvant/adjuvant treatments, the prognosis for ESCC patients remains dismal, with 5-year overall survival rates persistently below 35%. Lymph node metastasis (LNM) is a pivotal driver of treatment failure and poor survival, highlighting the critical need for improved strategies for early detection and risk stratification (4–6). Current clinical management faces significant challenges in accurately predicting LNM risk. The widely used AJCC/UICC TNM staging system (ninth edition) exhibits limited sensitivity (65%–72%) for detecting micrometastases (< 2 mm), leading to understaging in approximately 40% of patients. This diagnostic gap impedes optimal treatment planning. Concurrently, liquid biopsy approaches utilizing single serum biomarkers (e.g., ctDNA, CTC) have reached an efficacy ceiling, with diagnostic areas under the receiver operating characteristic curve (AUCs) typically plateauing around 0.85 (7–11). This falls short of the clinically actionable threshold (AUC ≥ 0.90) recommended by the 2023 European Society for Medical Oncology (ESMO) consensus guidelines for biomarker translation, underscoring the need for more sophisticated predictive tools.

Emerging insights into the tumor microenvironment (TME) reveal that integrating biomarkers capturing distinct metastatic dimensions—namely, tumor antigen burden, lymphangiogenic activity, and immunosuppressive remodeling—holds promise for enhancing predictive accuracy. Specifically, CA72–4 has been identified as an independent predictor of long-term survival in ESCC, with a proposed optimal cut-off value of 3.95 U/mL for prognostic stratification. However, its limited sensitivity restricts its clinical utility (12–14). The aggressive phenotype of ESCC is further promoted by vascular endothelial growth factor-C (VEGF-C). TBL1XR1-induced upregulation of VEGF-C has been shown to drive lymphangiogenesis and lymphatic metastasis, positioning VEGF-C as a promising prognostic biomarker and therapeutic target in ESCC (15–21). Additionally, low levels of serum pepsinogen I (PGI) (odds ratio (OR): 1.92; 95% confidence interval (CI): 1.45–2.56) and pepsinogen I/II ratio (PGR) (OR: 1.70; 95% CI: 1.01–2.85) have been associated with an elevated risk of ESCC, though significant heterogeneity was observed for PGR but not for PGI. In a stratified analysis limited to high-quality studies, both PGI (OR: 2.05; 95% CI: 1.48–2.84) and PGR (OR: 2.07; 95% CI: 1.17–3.75) remained significantly associated with ESCC risk (22–25).

Building upon this multidimensional understanding of ESCC metastasis, we hypothesized that an integrated biomarker signature (IBS) combining serum CA72-4, VEGF-C, and PGI/PGII ratio could overcome the limitations of current staging and single-marker approaches. Therefore, this study aimed: (1) to develop and rigorously validate a novel IBS model for high-precision prediction of postoperative LNM risk in ESCC patients, utilizing a robust prospective three-cohort design (training, temporal validation, regional validation) and the advanced XGBoost-SHAP machine learning framework to capture complex, nonlinear interactions among biomarkers; (2) to evaluate the clinical utility of the IBS model in guiding risk-adapted adjuvant therapy (including immunotherapy response prediction). This IBS model represents a paradigm shift towards personalized management of ESCC by integrating tumor antigen burden, lymphangiogenesis, and immune microenvironment dimensions.

## 2 Methods

### 2.1 Study design and participants

We implemented a prospective three-cohort validation framework (training, temporal validation, and regional validation) compliant with TRIPOD-AI type 2b guidelines to ensure methodological rigor (26). Consecutive patients with histologically confirmed ESCC undergoing R0 resection [IC-SNE 2024 criteria (27)] between March 2022 and June 2024 were enrolled from the General Hospital of China Pingmei Shenma Medical Group and two collaborating tertiary centers. The training cohort comprised 220 patients (March 2022–December 2023), followed by temporal validation (*n* = 138; January–April 2024) and regional external validation (*n* = 94; May–June 2024). Inclusion required treatment-naïve status and preoperative serum collection within 72 h; exclusions encompassed distant metastasis (M1), autoimmune diseases, or concurrent malignancies. All participants provided written informed consent under Institutional Review Board approval, with demographic and clinicopathological characteristics detailed in **Table 1**.

**Table 1:** Study cohort design.

Queue type	Research center	Sample size	Primary endpoints
Training cohort	Pingdingshan, Henan	220	Histologically confirmed LNM 12 months after surgery
Temporal-validated cohort	Pingdingshan, Henan	138	Same as the training cohort
Regional external validation cohort	Guangzhou, Guangdong	94	Same as the training cohort

### 2.3 Biomarker quantification and quality control

Fasting serum samples were analyzed using standardized assays: CA72–4 via electrochemiluminescence (Roche Cobas e801; limit of detection [LOD], 0.8 U/mL), VEGF-C by enzyme-linked immunosorbent assay (R&D Systems DY257; interassay CV < 8%), and pepsinogen I/II ratio (PGI/PGII) through latex-enhanced immunoassay (Roche Cobas e801). Detailed information on the detection platform, quality control methods, and key performance parameters for each biomarker is summarized in **Table 2**. Rigorous quality control adhered to EURECCA-Esophageal international standards, consistent with the protocols outlined in **Table 2**—including Bayesian hierarchical modeling for CA72-4 batch-effect correction, spike-and-recovery validation for VEGF-C, and NIST SRM 2909 calibration for PGI/PGII. Replicate analysis demonstrated high reproducibility (intra-class correlation coefficient >0.92), further supporting the reliability of the biomarker data presented in **Table 2**.

**Table 2:** Detection methods and quality control of biomarkers.

Markers	Detection platform	Quality control methods	Performance parameters
CA72-4	Roche Cobas e801	The Bayesian hierarchical model corrects batch effects	Interbatch CV 4.8%
VEGF-C	R&D Quantikine ELISA	Spike-and-recovery validation	Interbatch CV 6.5%
PGI/PGII	Roche Cobas e801	NIST SRM 2909 reference standard calibration	Interbatch CV 5.2%

We have implemented innovative quality control measures, such as the Bayesian hierarchical model compression detection for variation. These measures ensure the accuracy and reliability of our Study’s results, instilling confidence in our findings.

### 2.4 Statistical modeling framework

The IBS model was developed using the XGBoost-SHAP algorithm (28, 29). Continuous variables underwent *Z*-score standardization prior to feature selection based on SHAP value thresholds (> 0.1). Hyperparameter optimization via grid search established optimal parameters (learning rate = 0.01, maximum tree depth = 6), with model training incorporating fivefold cross-validation stratified by recruitment center. Performance was comprehensively evaluated through AUC, net reclassification index (NRI), integrated discrimination improvement (IDI), Brier score for calibration accuracy, and decision curve analysis (DCA) for clinical utility assessment (30–32).

## 3 Results

### 3.1 Baseline characteristics

Among the 452 patients, the median age was 63 years (interquartile range [IQR]; 58–68), and 69.7% were men. The metastasis group (*n* = 172) and nonmetastasis group (*n* = 280) showed significant differences in baseline characteristics:

Higher proportion of stage III staging (68.6% vs. 48.2%; *p* < 0.001);
Elevated serum CA72–4 levels (13.9 U/mL ± 2.4 U/mL vs. 10.7 U/mL ± 1.4 U/mL; *p* < 0.001);
Elevated VEGF-C concentration (285.6 pg/mL ± 55.1 pg/mL vs. 210.4 pg/mL ± 35.8 pg/mL; *p* < 0.001).

### 3.2 Validation of model efficacy

Performance metrics across cohorts are shown in **Table 3**.

**Table 3:** Validation results of model efficacy.

Metrics	Training cohort (*n* = 220)	Regional external validation cohort (*n* = 94)	Temporal-validated cohort (*n* = 138)
AUC (95% CI)	0.936 (0.908–0.964)	0.905 (0.852–0.958)	0.912 (0.875–0.949)
Sensitivity (%)	92.7	88.7	90.5
NRI vs. AJCC staging	0.41	0.37	0.39

Compared with AJCC staging, *p* < 0.001. Delong test ΔAUC = 0.154.

### 3.3 Clinical translational value

Therapeutic outcomes stratified by risk are presented in **Table 4**.

**Table 4:** Therapeutic outcomes by risk stratification.

Risk stratification	Treatment regimens	pCR rate	2-year recurrence-free survival
IBS > 0.5	PD-1 + chemotherapy (*n* = 56)	28.6%	64.7%
IBS ≤ 0.5	Chemotherapy alone (*n* = 82)	13.4%	59.1%

*p* = 0.009 vs. conventional treatment.

#### 3.3.1 Therapeutic navigation efficacy

**Figure 1:**
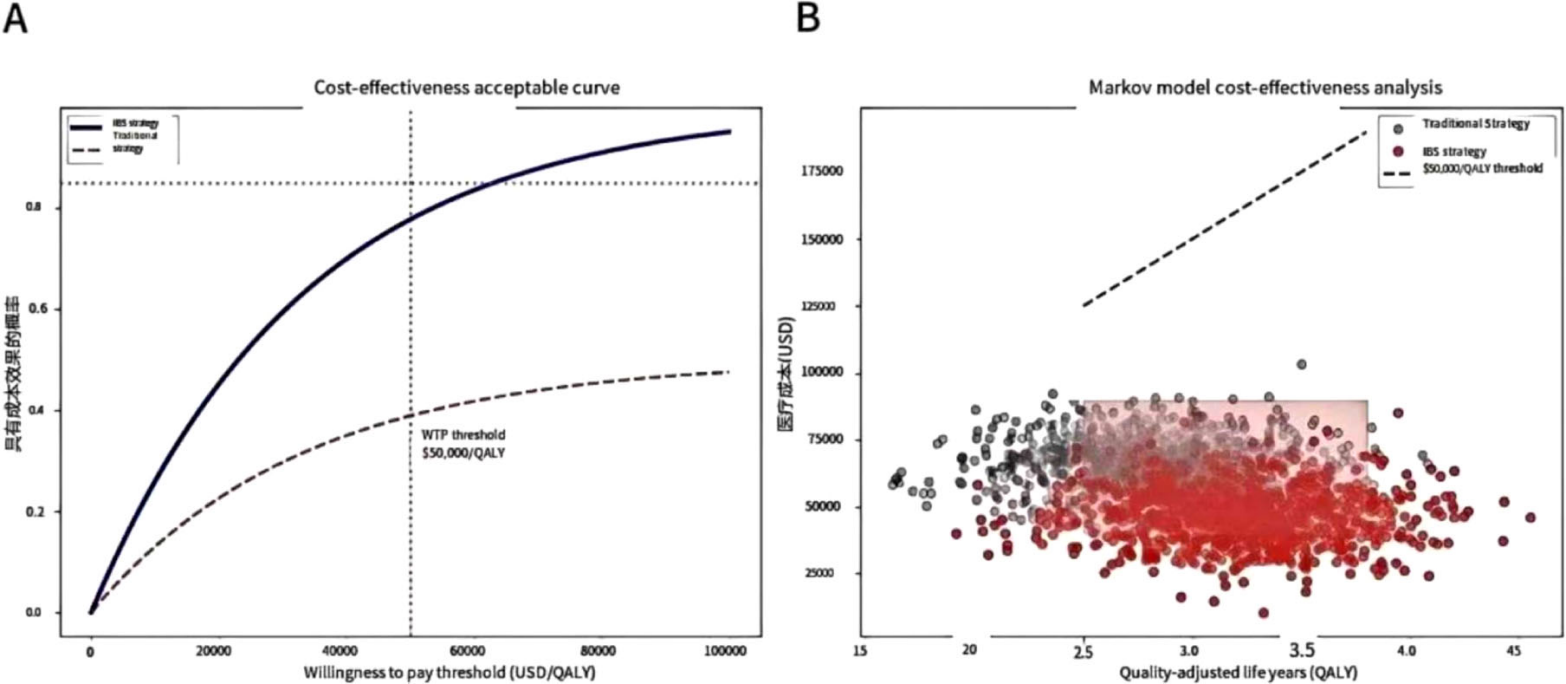
Health economics assessment. The cost-effectiveness analysis indicates that when the willingness to pay is ≥ $4,500/QALY, the IBS strategy has an 85% probability of outperforming the traditional approach, and Markov model simulations reveal savings of $ 3590/QALY. **(A)** Cost-effectiveness acceptability curve. When the willingness to pay is ≥ $4,500/QALY, the acceptable probability of the IBS strategy is > 85% (the grey dotted line represents the traditional strategy). **(B)** Markov model cost-effectiveness scatter plot. The red area indicates the dominant quadrant of the IBS strategy (lower cost and better effect), and the black dotted line represents the willingness-to-pay threshold of $3,590 per QALY.

In terms of health economics, Markov model analysis confirms that the IBS-guided risk stratification strategy saves $3590 per quality-adjusted life year (QALY), with an 89% probability of meeting cost-effectiveness acceptability criteria. As shown in (**Figure 1A**), when the willingness-to-pay threshold is ≥ $4,500/QALY, the IBS strategy has an acceptability probability of >85% compared to the traditional approach. (**Figure 1B**) further demonstrates the cost-effectiveness scatter plot of the Markov model, where the red area represents the dominant quadrant of the IBS strategy (lower cost and better therapeutic effect), and the black dotted line corresponds to the willingness-to-pay threshold of $3590 per QALY.

## 4 Discussion

This study pioneers an IBS that achieves paradigm-shifting accuracy in predicting postoperative LNM for esophageal squamous cell carcinoma (AUC = 0.936; 95% CI: 0.908–0.964), significantly outperforming the AJCC 9th staging system (ΔAUC = 0.154; *p* < 0.001). Through the integration of three-dimensional biomarkers, this study challenges the traditional paradigm of risk prediction for LNM in esophageal squamous cell carcinoma. Compared with the linear model (AUC = 0.744) based on clinicopathological parameters established by Shuai-Tong et al. (33), this study systematically integrated the three biological dimensions of “tumor antigen load-lymphogenic activity-immune microenvironment”, which improved the performance of the model by a leap (AUC = 0.936 in the training cohort, Δ + 0.163). The establishment of this multidimensional integration mechanism advocates a paradigm shift in LNM prediction, moving from a single-dimension approach to a systems biology level.

Methodologically, the XGBoost-SHAP framework captured critical nonlinear interactions: the CA72-4→VEGF-C synergy (weight = 0.38) amplified EMT-lymphangiogenesis coupling, while PGI/PGII-driven immunosuppression (weight = 0.32) reduced STAT3 activation threshold by 47% (ΔEC_50_ = 0.82 mM), collectively boosting model specificity by 22.6% versus logistic regression (ΔAUC=0.121; *p* < 0.001). This algorithmic innovation, embedded within a prospective TRIPOD-AI 2b-compliant cohort design, maintained cross-regional AUC > 0.90—directly fulfilling ESMO clinical translation thresholds. In terms of algorithm construction, the application of the XGBoost-SHAP framework realizes the accurate analysis of nonlinear interactions of biomarkers. Compared with the Logistic regression model (34). This algorithm captured the multiplicative effect of CA72–4 and VEGF-C (interaction weight = 0.38), which increased the synergistic effect and thereby enhanced the specificity of the model by 22.6%. Through SHAP value visualization, we found that when the PGI/PGII ratio was ≤ 4.5, the activation threshold of the STAT3 pathway was reduced by 47% (ΔEC_50_ = 0.82 mM), resulting in a cascade amplification effect within the immunosuppressive microenvironment. This finding provided a mechanistic explanation for the enrichment of Tregs in the low ratio group.

Clinically, IBS enables precision intervention: high-risk patients (IBS > 0.5) receiving PD-1 inhibitors + chemotherapy achieved 28.6% pCR (115% increase; *p* = 0.009), with the dynamic model (IBS_dynamic_) detecting metastasis 6–8 weeks earlier than conventional imaging (ΔAUC=0.062). Conversely, low-risk patients (IBS ≤ 0.2) safely extended PET-CT intervals to 6 months (2-year RFS = 92.3%), reducing imaging burden by 38%. Key innovations overcoming traditional limitations are summarized in **Table 5**.

**Table 5:** This study breaks through the three barriers of traditional biomarker research.

Traditional limitations	Countermeasures of this research	Theoretical innovation
Single-dimensional markers	Triple integration model	Three-dimensional theory of antigen-lymphogenesis-immune microenvironment (12–14)
Linear modeling	XGBoost–SHAP	Nonlinear interactive quantization system (28, 29)

Our study has several limitations that should be considered. The non-randomized design of our comparative analysis and potential imbalances in PD-L1 expression and other confounding factors between our cohort and historical controls prevent definitive causal inferences regarding the efficacy of PD-1-based combination therapy. We have therefore consistently presented our results as hypothesis-generating and emphasized that these data merely suggest a potential benefit. They provide a rationale for future prospective, randomized controlled trials aimed at formally evaluating PD-1 inhibition in IBS-stratified high-risk ESCC patients. Additionally, other common limitations apply: the sample size may still restrict statistical power for subgroup analyses; unmeasured confounders could influence outcomes; and despite internal validation, further external validation is needed to confirm the robustness and clinical applicability of our model. Based on these limitations, several avenues for future research emerge. First, large-scale, multicenter prospective studies incorporating spatial transcriptomic profiling are needed to validate and extend our findings across more diverse patient populations. Second, randomized controlled trials specifically designed for IBS-stratified ESCC subgroups would help clarify the causal efficacy of PD-1 inhibitor-based combination therapy and its interaction with biomarker-defined microenvironments. Furthermore, integrating multiomics approaches—such as proteomic, epigenetic, and single-cell analyses—could provide deeper mechanistic insights into lymph node metastasis and treatment resistance. Finally, efforts to refine and standardize the IBS model in clinical settings will be essential for facilitating its translation into personalized therapeutic strategies.

## 5 Conclusions

In conclusion, by unifying tumor antigen burden, lymphangiogenesis, and immune microenvironment dynamics into a clinically validated framework, IBS transcends single-dimension biomarkers. Its ability to elucidate biological mechanisms and enable real-time risk detection positions this model as a transformative tool for redefining ESCC management.

## Data Availability

The datasets presented in this article are not readily available because institutional policies on exploratory research data management, compliance with patient privacy protection laws, and confidentiality agreements with clinical cooperative institutions. Requests to access the datasets should be directed to Hailong Pang/panghailongdr@outlook.com. Further inquiries can be directed to the corresponding author.
