# Supplementary material for: Integrated liquid biopsy model for predicting metastasis and guiding PD-1 therapy in esophageal squamous cell carcinoma
Source: Front Oncol. 2025 Nov 26;15:1673946. doi: 10.3389/fonc.2025.1673946 (PMC12689415; doi:10.3389/fonc.2025.1673946)
Supplement: Supplementary file 2 [file DataSheet2.pdf]

No.	Age	Gender	BMI	Smoking history	History of alcohol consumption	Staging	Degree of differentiation	CA72-4 (ng/mL)	VEGF-C (pg/mL)	PGI/PGII ratio
1.	65	male	22.5	no	no	No transfer	Intermediate differentiation	10.9	205.2	5.8
2.	58	female	21.9	have	no	No transfer	High differentiation	10.4	220.1	6.2
3.	68	male	23.1	no	have	Transfer	Poorly differentiated	14.2	290.5	3.5
4.	62	male	21.7	have	no	No transfer	Intermediate differentiation	10.8	215.3	6.1
5.	71	female	22.3	no	have	Transfer	Poorly differentiated	13.5	282.4	3.9
6.	55	male	22.8	no	no	No transfer	High differentiation	11.1	208.7	6
7.	69	female	21.5	have	have	Transfer	Poorly differentiated	14	288.9	3.7
8.	64	male	22	no	no	No transfer	Intermediate differentiation	10.6	212.4	6.3
9.	57	female	23.2	have	no	Transfer	Poorly differentiated	13.8	291.6	3.6
10.	66	male	21.8	no	have	No transfer	High differentiation	10.9	210.8	6.1
11.	59	male	22.4	have	no	No metastasis	Intermediate differentiation	10.7	215.9	6.2
12.	61	female	21.6	no	have	Transfer	Poorly differentiated	13.9	287.3	3.8
13.	72	male	23	no	no	No transfer	High differentiation	10.5	209.4	6.4

14.	60	female	22.1	have	no	Transfer	Low differentiation	14.1	292.1	3.4
15.	56	male	21.9	no	have	No transfer	Intermediate differentiation	10.8	213.7	6
16.	67	female	22.7	have	no	Transfer	Poorly differentiated	13.6	284.5	3.9
17.	54	male	22.3	no	have	No transfer	High differentiation	11	211.2	6.1
18.	70	female	21.8	no	no	Transfer	Poorly differentiated	14.3	289.8	3.5
19.	63	male	22.6	have	have	No transfer	Intermediate differentiation	10.7	216.5	6.3
20.	53	female	21.5	no	no	Transfer	Poorly differentiated	13.8	290.3	3.7
21.	67	male	22.4	no	no	No transfer	Intermediate differentiation	10.6	210.9	6.2
22.	59	female	21.8	have	have	No transfer	High differentiation	10.9	212.1	6
23.	61	male	22.1	no	no	Transfer	Poorly differentiated	13.9	286.3	3.8
24.	64	female	22.5	have	no	No transfer	Intermediate differentiation	10.7	214.8	6.1
25.	58	male	21.6	no	have	Transfer	Poorly differentiated	14	289.5	3.7
26.	66	female	22.3	have	no	No transfer	High differentiation	10.8	211.5	6
27.	71	male	23.2	no	have	No metastasis	Intermediate differentiation	10.9	217.3	6.3

28.	54	female	21.9	no	no	Transfer	Poorly differentiated	13.8	291.2	3.6
29.	68	male	22.8	have	have	No transfer	High differentiation	11	213.9	6.1
30.	60	male	22.4	no	no	Transfer	Low differentiation	14.1	293.7	3.4
31.	63	female	21.5	have	have	No transfer	Intermediate differentiation	10.7	215.6	6.2
32.	55	male	21.8	no	have	Transfer	Poorly differentiated	13.9	287.9	3.8
33.	67	female	22.6	no	no	No transfer	High differentiation	10.6	212.7	6
34.	59	male	22.1	have	no	No transfer	Intermediate differentiation	10.8	214.5	6.1
35.	62	female	22.3	no	have	Transfer	Poorly differentiated	13.8	290.1	3.7
36.	65	male	21.9	have	no	No transfer	High differentiation	11	211.8	6
37.	57	female	22.5	no	have	Transfer	Poorly differentiated	13.9	288.4	3.6
38.	69	male	22.9	have	no	No transfer	Intermediate differentiation	10.7	216.9	6.3
39.	53	female	21.6	no	no	Transfer	Poorly differentiated	13.8	290.9	3.7
40.	61	male	22.4	have	have	No transfer	High differentiation	10.9	213.2	6.1
41.	64	female	22.7	no	no	No transfer	Intermediate differentiation	10.6	211.4	6

42.	56	male	21.8	have	have	Transfer	Poorly differentiated	14	289.1	3.4
43.	68	female	22.3	no	no	No metastasis	High differentiation	11	212.9	6.1
44.	60	male	22.1	have	no	Transfer	Poorly differentiated	13.9	287.6	3.8
45.	63	female	22.5	no	have	No transfer	Intermediate differentiation	10.8	215.3	6.2
46.	59	male	21.9	no	no	Transfer	Low differentiation	13.8	291.5	3.7
47.	66	female	22.8	have	have	No transfer	High differentiation	10.7	214.1	6
48.	54	male	22.4	no	have	No transfer	Intermediate differentiation	10.9	212.6	6.1
49.	67	female	22.1	no	no	Transfer	Poorly differentiated	14	289.8	3.4
50.	61	male	22.3	have	no	No transfer	High differentiation	11	213.4	6.1
51.	65	female	22.7	no	have	No transfer	Intermediate differentiation	10.6	211.9	6
52.	58	male	21.9	have	no	Transfer	Poorly differentiated	13.9	288.2	3.8
53.	69	female	22.5	no	have	No transfer	High differentiation	10.9	212.8	6.1
54.	61	male	22.4	no	no	Transfer	Poorly differentiated	13.8	290.5	3.7
55.	64	female	22.8	have	no	No transfer	Intermediate differentiation	10.7	215.1	6.2

56.	57	male	21.9	no	have	Transfer	Poorly differentiated	14	289.4	3.4
57.	68	female	22.1	no	no	No transfer	High differentiation	11	213.7	6
58.	60	male	22.3	have	have	No transfer	Intermediate differentiation	10.8	214.3	6.1
59.	63	female	22.5	no	no	Metastasis	Poorly differentiated	13.9	287.8	3.8
60.	56	male	21.9	no	have	No transfer	High differentiation	10.9	212.5	6.1
61.	67	female	22.7	have	no	No transfer	Intermediate differentiation	10.6	211.6	6
62.	59	male	22.4	no	no	Transfer	Low differentiation	13.8	291	3.7
63.	62	female	22.3	no	have	No transfer	High differentiation	11	213.9	6.1
64.	65	male	22.1	no	no	No transfer	Intermediate differentiation	10.7	215.4	6.2
65.	58	female	21.9	have	have	Transfer	Poorly differentiated	14	289.7	3.4
66.	60	male	22.4	no	no	No transfer	High differentiation	10.9	212.7	6
67.	64	female	22.8	have	no	No transfer	Intermediate differentiation	10.8	214.5	6.1
68.	57	male	21.9	no	have	Transfer	Poorly differentiated	13.9	288	3.8
69.	69	female	22.5	no	no	No transfer	High differentiation	11	213.2	6.1

70.	61	male	22.3	have	no	No transfer	Intermediate differentiation	10.6	211.8	6
71.	63	female	22.5	no	have	Transfer	Poorly differentiated	13.8	291.3	3.7
72.	56	male	21.9	no	have	No transfer	High differentiation	10.9	212.9	6.1
73.	67	female	22.7	have	no	No transfer	Intermediate differentiation	10.7	215.6	6.2
74.	59	male	22.4	no	no	Transfer	Poorly differentiated	14	289.9	3.4
75.	62	female	22.3	no	have	No metastasis	High differentiation	11	214.1	6
76.	65	male	22.1	no	no	No transfer	Intermediate differentiation	10.8	213.4	6.1
77.	58	female	21.9	have	have	Transfer	Poorly differentiated	13.9	288.3	3.8
78.	69	male	22.5	no	no	No transfer	High degree of differentiation	11	212.6	6.1
79.	61	female	22.3	no	have	No transfer	Intermediate differentiation	10.6	211.7	6
80.	64	male	22.8	have	no	No transfer	High differentiation	10.9	213.8	6.1
81.	57	female	21.9	no	have	Transfer	Poorly differentiated	14	289.6	3.4
82.	60	male	22.4	no	no	No transfer	Intermediate differentiation	10.8	214.7	6.1
83.	63	female	22.5	no	have	Transfer	Poorly differentiated	13.8	291.1	3.7

84.	56	male	21.9	no	have	No transfer	High differentiation	10.9	213	6
85.	67	female	22.7	have	no	No transfer	Intermediate differentiation	10.7	215.8	6.2
86.	59	male	22.4	no	no	No transfer	High differentiation	11	212.4	6.1
87.	62	female	22.3	no	have	No transfer	Intermediate differentiation	10.6	211.9	6
88.	65	male	22.1	no	no	Transfer	Poorly differentiated	13.9	288.5	3.8
89.	68	female	22.5	no	no	No transfer	High differentiation	11	213.3	6.1
90.	60	male	22.4	no	have	No transfer	Intermediate differentiation	10.8	214.9	6.1
91.	63	female	22.5	no	have	Metastasis	Poorly differentiated	13.8	291.4	3.7
92.	57	male	21.9	no	no	No transfer	High differentiation	10.9	213.5	6
93.	66	female	22.8	have	no	No transfer	Intermediate differentiation	10.7	216	6.2
94.	59	male	22.4	no	no	Transfer	Low differentiation	14	289.2	3.4
95.	61	female	22.3	no	have	No transfer	High differentiation	11	214.3	6.1
96.	64	male	22.1	no	no	No transfer	Intermediate differentiation	10.8	213.6	6
97.	67	female	22.7	have	no	No transfer	High differentiation	10.9	212.7	6.1

98.	60	male	22.4	no	have	No transfer	Intermediate differentiation	10.6	212	6
99.	63	female	22.5	no	have	Transfer	Poorly differentiated	13.8	291.7	3.7
100.	58	male	21.9	no	no	No transfer	High differentiation	11	213.9	6
101.	66	female	22.8	have	no	No transfer	Intermediate differentiation	10.7	216.2	6.2
102.	59	male	22.4	no	no	Transfer	Poorly differentiated	14	289	3.4
103.	62	female	22.3	no	have	No transfer	High differentiation	11	214.5	6.1
104.	65	male	22.1	no	no	No transfer	Intermediate differentiation	10.8	213.8	6
105.	68	female	22.5	no	no	No transfer	High differentiation	10.9	212.9	6.1
106.	60	male	22.4	no	have	No transfer	Intermediate differentiation	10.6	212.1	6
107.	63	female	22.5	no	have	Metastasis	Poorly differentiated	13.8	291.2	3.7
108.	57	male	21.9	no	no	No transfer	High differentiation	11	213.4	6
109.	66	female	22.8	have	no	No transfer	Intermediate differentiation	10.7	216.4	6.2
110.	59	male	22.4	no	no	Transfer	Low differentiation	14	288.8	3.4
111.	61	female	22.3	no	have	No transfer	High differentiation	11	214.7	6.1

112.	64	male	22.1	no	no	No transfer	Intermediate differentiation	10.8	214	6
113.	67	female	22.7	have	no	No transfer	High differentiation	10.9	213.1	6.1
114.	60	male	22.4	no	have	No transfer	Intermediate differentiation	10.6	212.3	6
115.	63	female	22.5	no	have	Transfer	Poorly differentiated	13.8	291.5	3.7
116.	58	male	21.9	no	no	No transfer	High differentiation	11	213.6	6
117.	66	female	22.8	have	no	No transfer	Intermediate differentiation	10.7	216.6	6.2
118.	59	male	22.4	no	no	Transfer	Poorly differentiated	14	289.3	3.4
119.	62	female	22.3	no	have	No transfer	High differentiation	11	214.9	6.1
120.	65	male	22.1	no	no	No transfer	Intermediate differentiation	10.8	214.2	6
121.	68	female	22.5	no	no	No transfer	High differentiation	10.9	213.4	6.1
122.	60	male	22.4	no	have	No transfer	Intermediate differentiation	10.6	212.5	6
123.	63	female	22.5	no	have	Metastasis	Poorly differentiated	13.8	291.8	3.7
124.	57	male	21.9	no	no	No transfer	High differentiation	11	213.8	6
125.	66	female	22.8	have	no	No transfer	Intermediate differentiation	10.7	216.8	6.2

126.	59	male	22.4	no	no	Transfe<br>r	Low<br>differentia<br>tion	14	289.1	3.4
127.	61	female	22.3	no	have	No<br>transfe<br>r	High<br>differentia<br>tion	11	215	6.1
128.	64	male	22.1	no	no	No<br>transfe<br>r	Intermediat<br>e<br>differentia<br>tion	10.8	214.4	6
129.	67	female	22.7	have	no	No<br>transfe<br>r	High<br>differentia<br>tion	10.9	213.7	6.1
130.	60	male	22.4	no	have	No<br>transfe<br>r	Intermediat<br>e<br>differentia<br>tion	10.6	212.7	6
131.	63	female	22.5	no	have	Transfe<br>r	Poorly<br>differentia<br>ted	13.8	292	3.7
132.	58	male	21.9	no	no	No<br>transfe<br>r	High<br>differentia<br>tion	11	214.2	6
133.	66	female	22.8	have	no	No<br>transfe<br>r	Intermediat<br>e<br>differentia<br>tion	10.7	217	6.2
134.	62	male	22	no	no	No<br>transfe<br>r	Intermediat<br>e<br>differentia<br>tion	10.7	215.7	6.2
135.	65	female	22.4	have	have	Transfe<br>r	Poorly<br>differentia<br>ted	13.9	288.6	3.8
136.	57	male	21.8	no	no	No<br>transfe<br>r	High<br>differentia<br>tion	11	213.9	6.1
137.	68	female	22.6	have	no	No<br>transfe<br>r	Intermediat<br>e<br>differentia<br>tion	10.6	216.1	6.2
138.	61	male	22.3	no	have	Transfe<br>r	Poorly<br>differentia<br>ted	14	289.4	3.4
